# Association between social support for mothers of patients with eating disorders and mothers’ active listening attitude: a cohort study

**DOI:** 10.1186/s13030-023-00262-9

**Published:** 2023-02-14

**Authors:** Fujika Katsuki, Atsurou Yamada, Masaki Kondo, Hanayo Sawada, Norio Watanabe, Tatsuo Akechi

**Affiliations:** 1 Department of Psychiatric and Mental Health Nursing, Nagoya City University Graduate School of Nursing, 1 Kawasumi, Mizuho-Cho, Mizuho-Ku, Nagoya, Japan; 2 Department of Psychiatry and Cognitive-Behavioral Medicine, Nagoya City University Graduate School of Medical Sciences, 1 Kawasumi, Mizuho-Cho, Mizuho-Ku, Nagoya, Japan; 3 Department of Psychiatry, Soseikai General Hospital, 101 Shimotoba, Hiroosa-Machi, Fushimiku, Kyoto Japan

**Keywords:** Caregiver, Eating disorders, Social support, Mother-patient relationship, Symptoms of eating disorders

## Abstract

**Background:**

Family members of patients with eating disorders, especially their mothers, experience heavy caregiving burdens associated with supporting the patient. We predict that increasing caregivers’ support will have a positive effect on their active listening attitudes, mental health, loneliness, and self-efficacy. This study aimed to investigate differences in mothers’ active listening attitudes, mental health, loneliness, and self-efficacy improvements between mothers who did and did not experience increased perceived social support.

**Main body:**

Participants were mothers of patients with eating disorders. Questionnaires for this cohort study were sent to the participants’ homes at three time points (baseline, 9 months, and 18 months). The Japanese version of the Social Provision Scale (SPS-10) was used to evaluate social support, the Active Listening Attitude Scale (ALAS) for listening attitude, the UCLA Loneliness Scale (ULS) for loneliness, the General Self-Efficacy Scale (GSES) for self-efficacy, the Beck Depression Inventory (BDI-II) for depression symptoms, and the K6 for psychological distress. An unpaired t-test was used to determine whether participants’ status differed between the groups that did and did not experience increased perceived social support. The mean age of the participants was 55.1 ± 6.7 (mean ± SD) years. The duration of their children’s eating disorders was 7.6 ± 5.5 years. The degree of improvement for each variable (active listening attitude, loneliness, self-efficacy, depressive symptoms, and mental health) was the difference in each score (ALAS, ULS, GSES, BDI-II, and K6) from T1 to T3. The degree of improvement in active listening attitude and loneliness was significantly greater in the improved social support group than in the non-improved social support group (*p* < 0.002 and *p* < 0.012, respectively).

**Conclusions:**

Our findings indicate that increasing mothers’ perceptions of social support will be associated with improving their active listening attitudes and loneliness.

## Background

Eating disorders are serious mental disorders associated with high levels of mortality and disability, physical and psychological morbidity, and impaired quality of life. The estimated standardized mortality ratios are 5.86 for anorexia nervosa (AN), 1.7 for bulimia nervosa, and 1.92 for eating disorders not otherwise specified [1]. Evidence of effective treatments for eating disorders in children and adolescents has been established [2]. However, a network meta-analysis of psychological interventions in adult AN outpatients reported no strong evidence to support the superiority or inferiority of any of the specific treatments recommended by clinical guidelines [3]. Even if there are evidence-based treatments for child and adolescent eating disorders, it is difficult to provide them to everyone who needs them.

With few effective treatments available, many families live with individuals with eating disorders who often engage in problematic behaviors such as suicidal behavior, obsessional thoughts, refusal of treatment, and behaviors concerning weight, body shape, and food for extended periods of time [4]. Family members who spend much of their time with the patient, especially mothers, suffer a heavy psychological burden. Although some studies have reported that caregivers do not experience high levels of distress, many studies have suggested that they experience high levels of depression and anxiety [5, 6].

Patients with eating disorders have been found to lack confidence in identifying their thoughts and feelings [7, 8]. Submissive behavior was significantly higher in patients with eating disorders than in healthy controls [9]. Some studies have identified a strong relationship between low levels of assertiveness and eating disorder psychopathology [10, 11]. It is believed that patients with eating disorders are unable to recognize and assert their thoughts and desires. Mothers’ good active listening ability may promote self-expression in patients with these characteristics. However, as mentioned above, mothers often experience high distress owing to the difficulty of responding to various symptoms and their involvement in eating disorder behaviors. Such situations make it difficult for mothers to relax and listen to patients well. Several studies have reported that caregiver support is associated with psychosocial stress and coping. Perceived high social support has been significantly associated with lower levels of depression among mothers of patients with eating disorders [12]. Some studies have reported that social support increases caregivers’ ability to cope [13, 14].

This cohort study aimed to investigate differences in mothers’ active listening attitudes, mental health, loneliness, and self-efficacy improvements between mothers who did and did not experience increased perceived social support.

## Methods

### Design

This was a longitudinal survey with assessments at three time points: baseline (Time 1 [T1]), 9 months (Time 2 [T2]), and 18 months (Time 3 [T3]).

### Participants

Participants were mothers of patients with eating disorders. Inclusion criteria were age between 30 and 85 years and being the mother of a child who met the patient inclusion criteria. The inclusion criteria for patients were age between 16 and 50 years and a clinical diagnosis of an eating disorder from a physician or psychiatrist in a hospital. Regarding caregivers, although we understand the fathers’ involvement is important for the treatment of eating disorders, this study focuses only mothers to ensure the homogeneity of the participants because it has been reported that mothers have a higher burden than fathers [15]. To recruit participants, we conducted lectures at four locations in Japan (i.e., Hokkaido, Chiba, Fukui, and Nagoya). These lectures included an introduction to the study. Participants were recruited between July 2017 and August 2018, with the last follow up in February 2020. Participants were provided with a leaflet explaining the study’s purpose and procedures. After receiving permission, we mailed the questionnaires to the participants’ homes. Participants were formally enrolled in the study only when their completed baseline questionnaires were returned. The questionnaires were mailed at three time points (i.e., at baseline, 9 months, and 18 months) and returned each time. The scales completed at each time point were the same. This study was approved by the Ethics Review Committee of Nagoya City University Graduate School of Medical Sciences, Japan (Ref: No 60–17-0001).

### Outcome measures

A shortened version of Social Provisions Scale (SPS-10) by Iapichino et al. was used to evaluate mothers’ perception of social support [16]. We created a Japanese version of the SPS-10 [17]. The SPS-10 consists of 10 items and retains the following five of the six original SPS subscales: attachment, social integration, reassurance of worth, reliable alliance, and guidance. The total SPS-10 score ranges from 10 to 40. A higher score indicates a stronger perception of social support. The General Self-Efficacy Scale (GSES) developed by Sakano et al. was used to assess mothers’ self-efficacy [18]. The higher the score, the higher the general self-efficacy. The Japanese version [19] of the University of California, Los Angeles Loneliness Scale (ULS), originally developed by Russell et al., was used to assess mothers’ loneliness [20]. The higher the score, the stronger the loneliness. The Active Listening Attitude Scale (ALAS) developed by Mishima et al. was used to assess mothers’ active listening attitude [21]. The higher the score, the better the listening attitude or skill. The Japanese version [22] of the Beck Depression Inventory—Second Edition (BDI-II), originally developed by Beck et al., was used to assess depression within the last two weeks [23]. The higher the score, the more severe the depressive symptoms. The Japanese version [24] of the Kessler Psychological Distress Scale (K6), originally developed by Kessler et al., was used to assess psychological distress [25]. The higher the score, the more severe the psychological distress.

### Statistical analysis

Descriptive data analysis was conducted by calculating mean scores, standard deviations, or rates (%). Correlations among the variables at T1 were examined using Pearson’s correlation coefficient. We examined changes in status between mothers who did and did not experience increased perceived social support. Mothers who experienced increased perceived social support comprised those whose SPS-10 scores increased from T1 to T3. Mothers who did not experience increased perceived social support consisted of participants whose SPS-10 scores remained the same or decreased from T1 to T3. We used an unpaired t-test to compare the two groups, with significance set at *p* < 0.05. Statistical analyses were performed using SPSS Statistics version 22.

## Results

### Participants’ characteristics

We mailed 85 sets of questionnaires to the participants’ homes, 72 of which were returned. The mean age was 55.1 ± 6.7 (mean ± SD) years. The duration for which their children had suffered from an eating disorder was 7.6 ± 5.5 years. Half of the participants cooperated with their partner in caring for the patient, and 68.4% were currently part of a family self-help group. Table 1 summarizes the participants’ baseline (T1) characteristics, and Table 2 summarizes the participants’ scores at all three time points. Correlations among the variables at T1 are summarized in Table 3. There were moderate or strong significant correlations between the scales.

**Table 1:** Participants’ baseline characteristics (*n* = 72)

Age, years, mean (SD)		55.1 (6.7)
Mother’s confirmation of patient being under medical care, years, mean (SD)		7.6 (5.5)
Mother’s experience with counseling, n (%)	Currently receiving	12 (15.8%)
	Received in the past	28 (36.8%)
	No	31 (40.8%)
Mother’s history of eating disorder, n (%)	Yes	2 (2.6%)
	No	65 (85.5%)
	Unknown	4 (5.3%)
Mother’s feeling of cooperation with the father to handle the patient, n (%)	Yes	38 (50.0%)
	No	8 (10.5%)
	Neither agreement nor denial	21 (27.6%)
	Not applicable	4 (5.3%)
Mother’s experience with joining a family self-help group, n (%)	Yes	52 (68.4%)
	Yes, in the past	14 (18.4%)
	No	4 (5.3%)

**Table 2:** Participants’ scores at all three time points and the reliability of each scale in the current study

			T1		T2		T3	
	Scale	Cronbach's α	Mean (SD)	n	Mean (SD)	n	Mean (SD)	n
Active listening attitude	ALAS	0.86	35.8 (7.1)	71	36.8 (6.6)	65	37.1 (7.3)	63
Social support	SPS-10	0.89	31.1 (4.6)	72	31 (5.7)	66	39.5 (10.4)	64
Loneliness	ULS	0.90	38.5 (9.2)	69	38.1 (9.6)	58	38.7 (10.0)	63
Self-efficacy	GSES	0.80	7.2 (3.8)	72	7.8 (3.7)	65	7.8 (3.9)	65
Depressive symptoms	BDI-II	0.92	14.1 (9.7)	72	12 (9.6)	64	11.3 (9.2)	65
Mental health	K6	0.86	6.9 (4.2)	71	6.1 (4.3)	66	5.9 (4.5)	64

*T1* Baseline, *T2* 9 months, and *T3* 18 months, *ALAS* Active Listening Attitude Scale, *SPS-10* The Social Provision Scale-10 item, *ULS* University of California, Los Angeles Loneliness Scale, *GSES* General Self-Efficacy Scale, *BDI-II* Beck Depression Inventory, *K6* Kessler Psychological Distress Scale

**Table 3:** Correlations among variables at T1

	SPS-10 (T1)	ALAS (T1)	ULS (T1)	GSES (T1)	BDI-II (T1)	K6 (T1)
SPS-10 (T1)	1					
ALAS (T1)	.321^**^	1				
ULS (T1)	-.794^**^	-.252^*^	1			
GSES (T1)	.312^**^	.443^**^	-.417^**^	1		
BDI-II (T1)	-.443^**^	-.275^*^	.466^**^	-.465^**^	1	
K6 (T1)	-.398^**^	-.381^**^	.362^**^	-.468^**^	.753^**^	1

*SPS-10* The Social Provision Scale-10 item, *ALAS* Active Listening Attitude Scale, *ULS* University of California, Los Angeles Loneliness Scale, *GSES* General Self-Efficacy Scale, *BDI-II* Beck Depression Inventory, *K6* Kessler Psychological Distress Scale

^*^P < 0.05, **P < 0.01

### Comparison between the improved social support group and non-improved social support group

The groups of mothers who did and did not experience increased perceived social support consisted of 26 and 38 participants, respectively. The mean age of the participants who experienced increased perceived social support was 54.6 ± 7.2, and their children’s eating disorder duration was 7.5 ± 6.2 years. The mean age of the participants who did not experience increased perceived social support was 55.3 ± 6.0, and their children’s eating disorder duration was 7.6 ± 4.4 years. The degree of improvement for each variable (active listening attitude, loneliness, self-efficacy, depressive symptoms, and mental health) was the difference in each score (ALAS, ULS, GSES, BDI-II, and K6) from T1 to T3. The degree of improvement in active listening attitude and loneliness was significantly greater in the improved social support group than in the non-improved social support group (*p* < 0.002 and *p* < 0.012, respectively; Table 4). No significant difference was found between the groups regarding the score differences from T1 to T2 (Table 4).

**Table 4:** Comparison of groups that did and did not experience increased perceived social support

Mothers who experienced increased perceived social support group					Mothers who did not experience increased perceived social support group				
		Mean	SD	n	Mean	SD	n	t value	*p* value
Improvement of Active listening attitude	ALAS (ΔT3-T1)	3.40	5.90	24	-1.00	4.70	37	3.22	0.002
Improvement of Loneliness	ULS (ΔT3-T1)	-1.60	5.59	25	1.70	4.70	37	-2.59	0.012
Improvement of Self efficacy	GSES (ΔT3-T1)	0.84	2.27	26	0.44	1.94	38	0.75	0.455
Improvement of Depressive symptoms	BDI-II (ΔT3-T1)	-4.50	7.40	26	-1.70	8.00	38	-1.39	0.167
Improvement of Mental health	K6 (ΔT3-T1)	-1.80	3.40	25	-0.51	4.53	37	-1.20	0.234
Improvement of Active listening attitude	ALAS (ΔT2-T1)	1.20	4.60	25	0.45	4.71	35	0.60	0.548
Improvement of Loneliness	ULS (ΔT2-T1)	1.90	6.20	21	0.18	5.97	33	1.01	0.314
Improvement of Self efficacy	GSES (ΔT2-T1)	0.12	1.87	25	0.83	2.03	36	-1.38	0.170
Improvement of Depressive symptoms	BDI-II (ΔT2-T1)	-2.64	7.73	25	-0.94	7.80	35	-0.38	0.408
Improvement of Mental health	K6 (ΔT2-T1)	-0.65	3.05	26	-0.88	4.10	35	0.24	0.810

*T1* Baseline, *T2* at 9 months, *T3* at 18 months, *ALAS* Active Listening Attitude Scale, *ULS* University of California, Los Angeles Loneliness Scale, *GSES* General Self-Efficacy Scale, *BDI-II* Beck Depression Inventory, *K6* Kessler Psychological Distress Scale

## Discussion

This study investigated differences in active listening attitude, mental health, loneliness, and self-efficacy after 18-months follow-up among mothers who did and did not experience increased perceived social support. Our findings suggest that increasing a mothers’ perceptions of social support may be associated with improving their active listening attitudes and loneliness. Because being a single parent and eating alone have been associated with the onset of eating disorders [26], the parents’ concern and listening to them may be important for eating disorder prevention and recovery.

Patients with eating disorders have been found to lack confidence in identifying their own thoughts and feelings and have low levels of assertiveness [10, 11]; if their mothers consistently listened well, they would be able to explain themselves without anxiety. Marcos reported significant positive correlations between informative support for patients with eating disorders, including listening, encouraging, and advising, and family self-concept as evaluated by the patient [27]. Family self-concept refers to patient-evaluated feelings such as “I feel more or less happy at home” and “parents would help with any type of problem.” Results from the above studies indicate that caregivers who listen and encourage patients help them feel safe and have peace of mind at home. If mothers of patients with eating disorders listen well, patients are able to use verbal communication more frequently; they may be able to avoid using eating disorder behaviors. These findings revealed that increasing mothers’ perceptions of social support may be associated with improving their active listening attitudes.

However, the mothers in this study showed worse mental health status at T1 (Table 2; the average BDI-II score of 14.1 ± 9.2 for our participants indicated mild depressive symptoms). Such mental exhaustion situations make it difficult for mothers to listen to patients. At T1 (Table 3), there were strong significant correlations between SPS-10 and ULS (*r* =—0.794) and moderate significant correlations between the SPS-10 and K6 (*r* = -0.407) and the SPS-10 and BDI-II (*r* = -0.463). These results highlight the importance of continuing support from professionals and self-help groups for the mothers of patients with eating disorders.

This study has several limitations. First, the sample size is small. This may have resulted in the small effects we observed, subsequently preventing the detection of significant differences among the studied variables. Second, we did not identify or differentiate between subgroups of eating disorders. Thus, we believe that future studies should distinguish patients by specific eating disorder; different types may evoke different outcomes among caregivers. However, the strength of our study was its cohort study design using 18-month follow-up data.

## Conclusion

Our main findings suggest that social support for mothers of patients with eating disorders is associated with improving their active listening attitudes. Therefore, it is important that professionals and self-help support groups continuously support not only patients with eating disorders but also their caregivers.

## Data Availability

The datasets used and/or analyzed in the current study are available from the corresponding author upon reasonable request.

## Abbreviations

ALAS: Active Listening Attitude Scale
BDI-II: Beck Depression Inventory
CFI: Comparative Fit Index
GSES: General Self-Efficacy Scale
K6: Kessler Psychological Distress Scale
RMSEA: Root Mean Square Error of Approximation
SEM: Structural Equation Modeling
SPS: The Social Provisions Scale
SPS-10: The Social Provisions Scale-10 item
ULS: University of California, Los Angeles Loneliness Scale
